# Laryngospasm-Induced Negative-Pressure Pulmonary Edema After Laparoscopic Appendectomy in a Young Adult: A Case Report

**DOI:** 10.7759/cureus.114853

**Published:** 2026-08-20

**Authors:** Mohammad Awawdeh, Ahmed Zareer, Ahmad Abdallah, Ilyas Awawdeh, Thaer Hamdan

**Affiliations:** 1 Department of Medicine, An-Najah National University, Nablus, PSE; 2 Department of Medicine, Palestine Polytechnic University, Hebron, PSE

**Keywords:** continuous positive airway pressure, laparoscopic appendectomy, laryngospasm, negative-pressure pulmonary edema, post-extubation hypoxemia, postoperative pulmonary edema

## Abstract

Negative-pressure pulmonary edema (NPPE) is an uncommon postoperative form of noncardiogenic pulmonary edema resulting from forceful inspiratory efforts against an obstructed upper airway. A 20-year-old man (62 kg, 172 cm; body mass index, 21.0 kg/m²) underwent laparoscopic appendectomy under general anesthesia. Preoperative oxygen saturation was 98% on room air. Immediately after extubation, laryngospasm occurred, followed by acute respiratory distress, oxygen desaturation to 74%, tachypnea at 35 breaths/min, and tachycardia at 125 beats/min. Arterial blood gas analysis showed pH 7.33, PaCO₂ 32 mmHg, and PaO₂ 53 mmHg. Chest radiography demonstrated bilateral patchy central air-space opacities, more pronounced in the right lung, without apparent cardiomegaly. High-sensitivity troponin T was within the reference range. NPPE secondary to post-extubation laryngospasm was diagnosed. Continuous positive airway pressure (CPAP) at 5 cmH₂O with supplemental oxygen was initiated, and a single 20-mg intravenous dose of furosemide was administered. Respiratory status progressively improved, and the patient was discharged in good condition on postoperative day 3. This case emphasizes rapid recognition of the laryngospasm-hypoxemia-pulmonary edema sequence and careful assessment of competing postoperative diagnoses.

## Introduction

Negative-pressure pulmonary edema (NPPE) is an acute noncardiogenic pulmonary edema caused by markedly negative intrathoracic pressure generated during forceful inspiration against an obstructed upper airway [1-3]. Post-extubation laryngospasm is a classic perioperative precipitant, particularly during emergence from general anesthesia [2-4]. The resulting pressure changes increase pulmonary vascular transmural pressure and favor rapid movement of fluid into the pulmonary interstitial and alveolar compartments; increased venous return, altered left ventricular loading, and stress on the alveolar-capillary interface may further contribute [1-4].

Clinical presentation is usually abrupt and may include hypoxemia, tachypnea, tachycardia, respiratory distress, and new pulmonary infiltrates [1-4]. Because aspiration pneumonitis, cardiogenic pulmonary edema, and perioperative fluid overload may present similarly, diagnosis depends heavily on the temporal relationship between upper-airway obstruction and respiratory deterioration [1-4]. NPPE after appendectomy has been reported previously [5,6]. We present a young adult who developed documented post-extubation laryngospasm followed by severe hypoxemia and radiographic pulmonary edema after laparoscopic appendectomy.

## Case presentation

A 20-year-old man weighing 62 kg and measuring 172 cm in height (body mass index, 21.0 kg/m²) presented with acute periumbilical and lower abdominal pain. Preoperative oxygen saturation was 98% on room air, and respiratory rate was 17 breaths/min. Pre-anesthetic assessment included airway, cardiovascular, and respiratory examination; review of medications and allergies; confirmation of fasting status; and review of relevant laboratory investigations.

**Figure 1:**
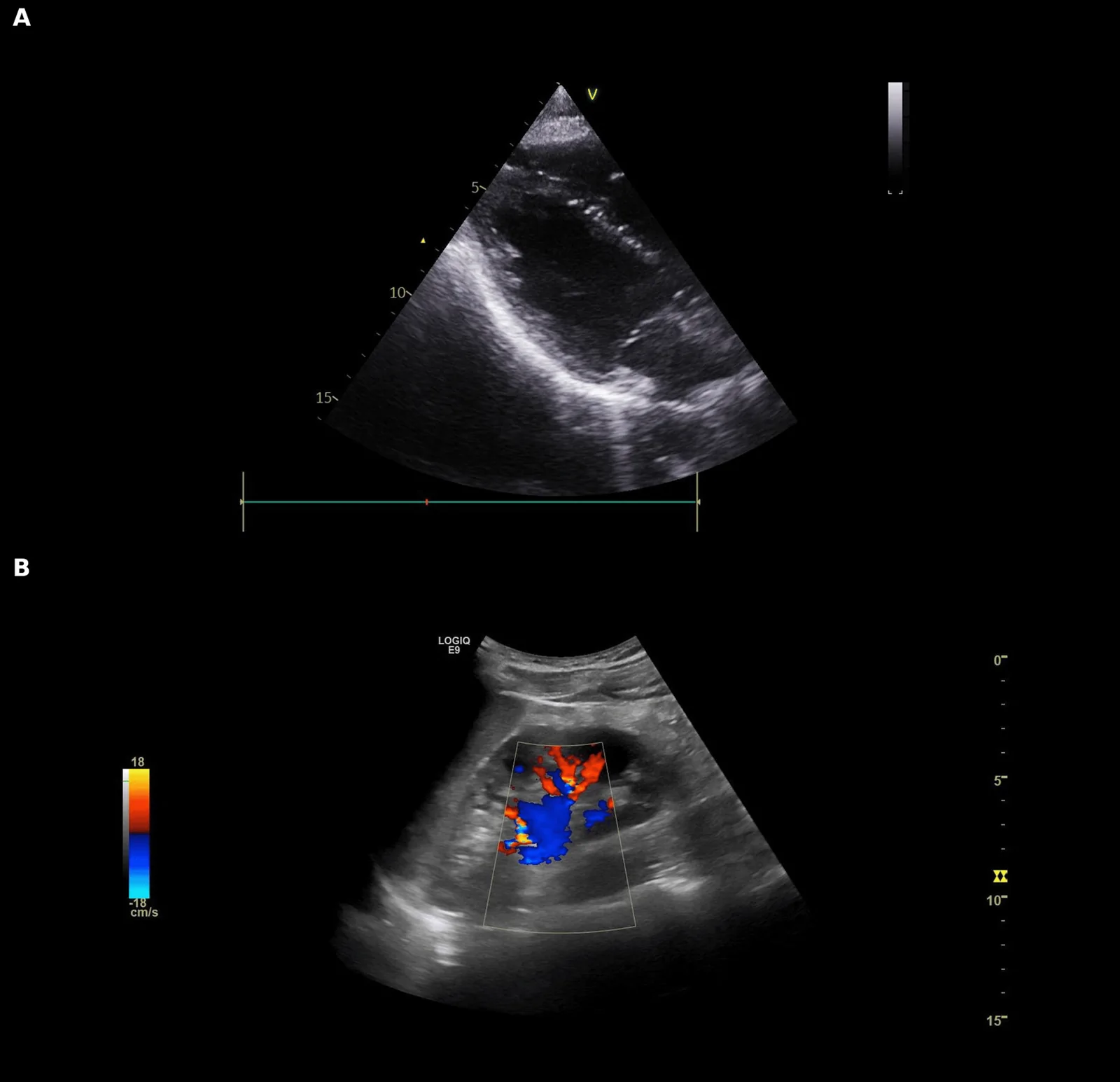
Right Renal Ultrasonography. (A) Grayscale image from the right renal examination demonstrating marked fluid distension of the pelvicalyceal system. (B) Color Doppler image from the same renal examination. The ultrasound report described severe right hydronephrosis with cortical thinning and no ureteric dilatation.

Abdominal ultrasonography demonstrated severe right hydronephrosis with marked dilatation of the renal pelvis and calyces, internal echoes/debris within the collecting system, cortical thinning, and no right ureteric dilatation. The right kidney measured approximately 13.5 cm in bipolar length, with reported cortical thickness of approximately 0.4 cm (Figure 1).

**Figure 2:**
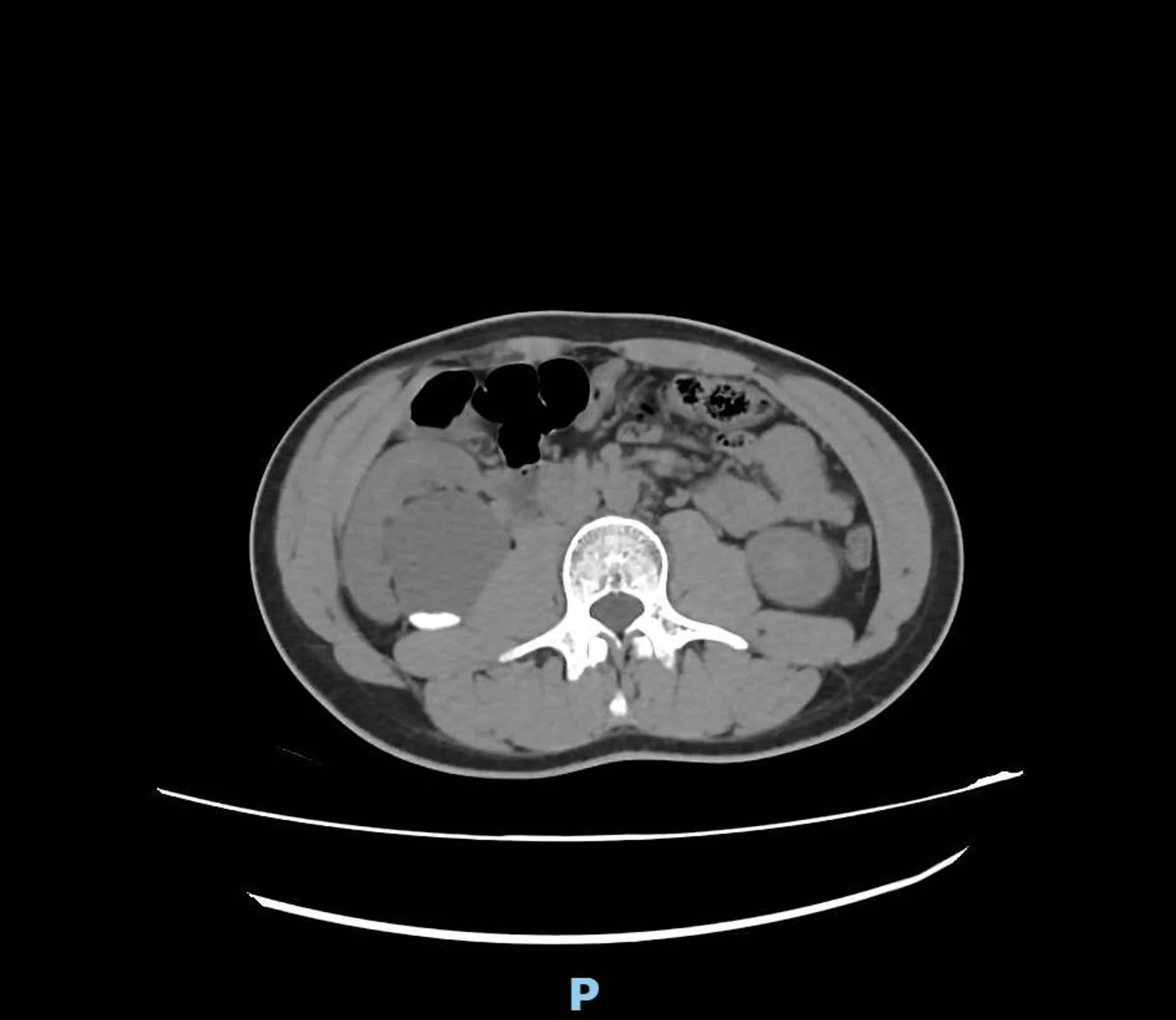
Right Renal Pelvicalyceal Dilatation With Renal-Pelvic Calculus. Contrast-enhanced axial CT image demonstrating marked right pelvicalyceal dilatation and a hyperdense renal-pelvic calculus. Associated cortical thinning and absence of ureteric dilatation contributed to the radiologic impression of pelviureteric junction stenosis.

Contrast-enhanced computed tomography showed a retrocecal appendix measuring up to 12 mm with surrounding fat stranding and adjacent fluid, consistent with acute retrocecal appendicitis. CT also demonstrated rotation of the right kidney, marked dilatation of the pelvicalyceal system, and moderate cortical thinning. A hyperdense renal-pelvic calculus measuring 2 × 2 × 0.6 cm with an attenuation of approximately 300 Hounsfield units was present, together with additional dependent lower-calyceal calculi (Figure 2). The right ureter was not dilated. The overall appearance was suggestive of pelviureteric junction stenosis, and the calculi were reported as non-obstructing.

Initial laboratory findings are summarized in Table 1. C-reactive protein was markedly elevated, with neutrophil predominance. Serum creatinine remained within the expected adult range, and high-sensitivity troponin T was within the laboratory reference range.

**Table 1:** Laboratory Findings at Presentation. Reference ranges are adult intervals used for interpretation of the reported laboratory results.

Laboratory test	Result	Reference range	Interpretation
White blood cell count	9.21 ×10³/µL	4.0-11.0 ×10³/µL	Within range
Neutrophils	93.7%	40-75%	High
C-reactive protein	53.8 mg/L	0-5 mg/L	High
Blood urea nitrogen	6.67 mg/dL	6-20 mg/dL	Within range
Serum creatinine	0.773 mg/dL	0.6-1.3 mg/dL	Within range
Total bilirubin	2.13 mg/dL	0.2-1.2 mg/dL	High
Direct bilirubin	0.776 mg/dL	0-0.3 mg/dL	High
Aspartate aminotransferase	21.8 U/L	10-40 U/L	Within range
Alanine aminotransferase	9.07 U/L	7-56 U/L	Within range
High-sensitivity troponin T	5.72 ng/L	0-22 ng/L	Within range

The patient underwent laparoscopic appendectomy under general anesthesia with endotracheal intubation and standard intraoperative monitoring. No clinically significant blood loss was recorded. Immediately after extubation, laryngospasm developed, followed by abrupt respiratory distress. Oxygen saturation decreased to 74%, respiratory rate increased to 35 breaths/min, and heart rate reached 125 beats/min. Arterial blood gas analysis demonstrated pH 7.33, PaCO₂ 32 mmHg, and PaO₂ 53 mmHg.

**Figure 3:**
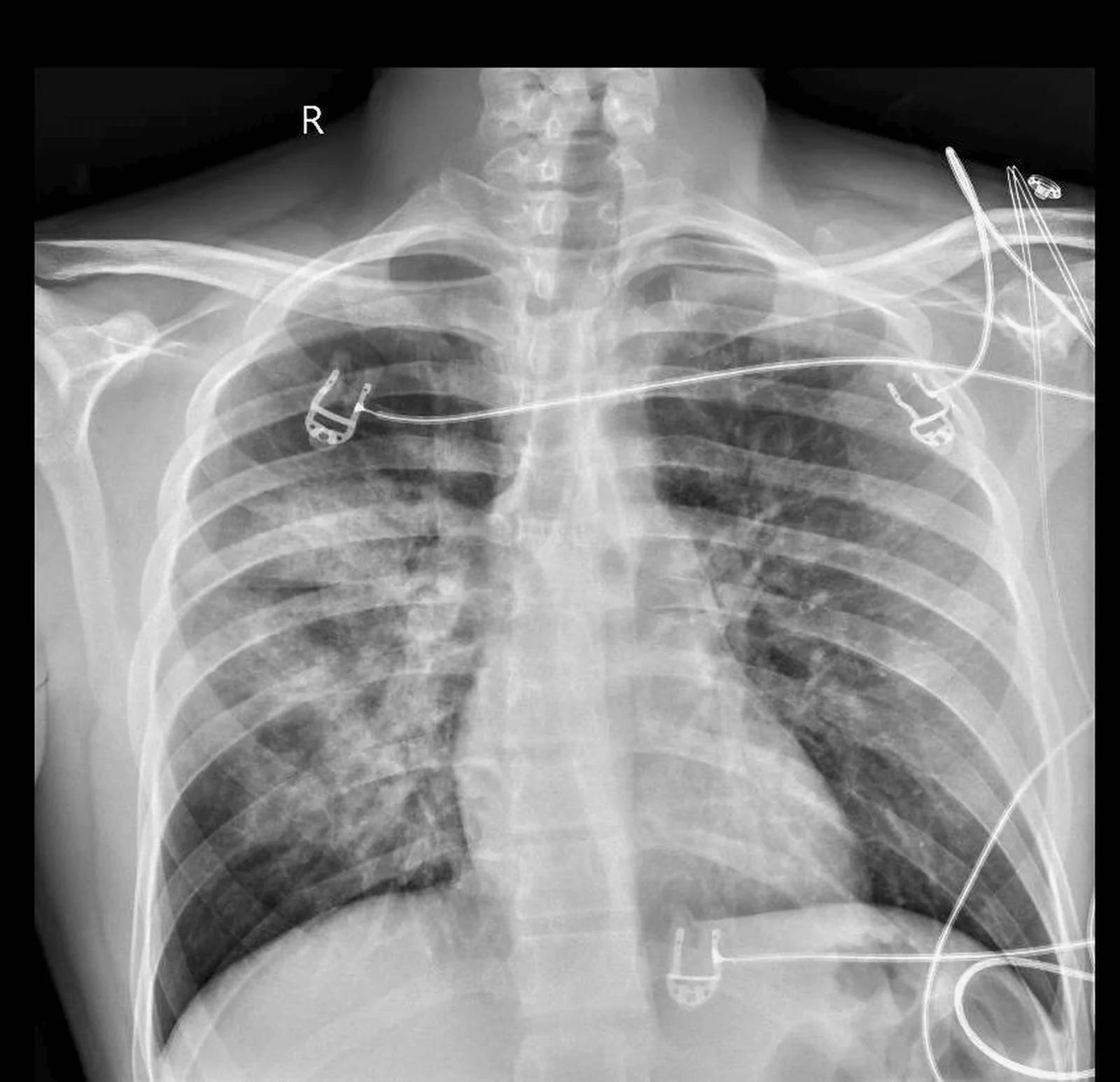
Post-Extubation Pulmonary Air-Space Opacities. Postoperative chest radiograph obtained after laryngospasm and acute respiratory deterioration, demonstrating bilateral patchy central/perihilar air-space opacities, substantially more pronounced in the right lung, without apparent cardiomegaly.

Postoperative chest radiography demonstrated bilateral patchy central/perihilar air-space opacities, substantially more pronounced in the right lung, without apparent cardiomegaly or a large pleural effusion (Figure 3). High-sensitivity troponin T was 5.72 ng/L, within the reference range. The immediate sequence of documented laryngospasm, profound hypoxemia, and new pulmonary edema supported the diagnosis of NPPE.

Continuous positive airway pressure (CPAP) was initiated at 5 cmH₂O with supplemental oxygen and continued for approximately 12 hours per day during postoperative monitoring. Furosemide 20 mg was administered intravenously once. Urine output and fluid status were monitored, with approximately 2,000 mL of urine recorded during the monitored period. Respiratory status progressively improved. At discharge, the patient was breathing comfortably without significant respiratory distress, tolerating oral intake, and ambulating appropriately. He was discharged home in good clinical condition three days after surgery.

## Discussion

The diagnostic strength of this case lies in its temporal sequence. The patient had normal baseline oxygenation, developed documented laryngospasm immediately after extubation, and then developed profound hypoxemia with radiographic pulmonary edema. This sequence is characteristic of NPPE, in which vigorous inspiratory effort against an obstructed airway generates markedly negative intrathoracic pressure and promotes pulmonary fluid translocation [1-4].

NPPE after appendectomy is not unique. Perez et al. reported NPPE immediately after extubation in a healthy 20-year-old man undergoing appendectomy [5]. Wong and Campbell described an 18-year-old man with post-extubation NPPE after appendectomy [6]. Rinaldi et al. reported post-extubation pulmonary edema after laparoscopic appendicectomy in another young patient [7], while Du et al. described an atypical post-appendectomy NPPE presentation accompanied by myocardial injury [8]. The present case should therefore not be framed as the first such report. Its educational value lies in the clearly documented laryngospasm-to-hypoxemia sequence, severe arterial hypoxemia, right-predominant radiographic pattern, and successful use of noninvasive positive-pressure support.

The principal differential diagnoses were aspiration pneumonitis, cardiogenic pulmonary edema, and perioperative fluid overload. Aspiration warranted particular consideration because the radiographic abnormalities were right-predominant; however, markedly asymmetric NPPE has been described after acute upper-airway obstruction [9]. Cardiogenic edema was less favored by the patient's young age, absence of apparent cardiomegaly, and normal high-sensitivity troponin T, although a normal troponin value alone cannot exclude cardiac dysfunction. Fluid overload was also considered. Renal function was preserved, and there was no clinically significant blood loss; however, the exact intraoperative intravenous fluid volume was unavailable, limiting complete assessment of perioperative fluid balance. Table 2 summarizes the case-specific differential diagnosis.

**Table 2:** Differential Diagnosis of Acute Post-Extubation Respiratory Deterioration. IV: intravenous; NPPE: negative-pressure pulmonary edema. Source: Authors' synthesis based on published NPPE literature [1-4,9].

Diagnosis	Why Considered	Case-Specific Interpretation
Negative-pressure pulmonary edema	Acute hypoxemia and new pulmonary edema after general anesthesia	Documented laryngospasm immediately after extubation, followed by abrupt severe hypoxemia and compatible chest radiography strongly favored NPPE.
Aspiration pneumonitis	Peri-anesthetic hypoxemia with right-predominant opacities may overlap	The immediate laryngospasm-to-hypoxemia sequence favored NPPE; aspiration should not be stated as excluded unless specifically documented.
Cardiogenic pulmonary edema	Pulmonary edema can have a cardiac origin	Young age, no apparent cardiomegaly, and normal high-sensitivity troponin T made acute myocardial injury less likely, but did not fully exclude cardiac dysfunction.
Perioperative fluid overload	Excess perioperative fluid can cause pulmonary edema	Renal function was preserved, and no significant blood loss occurred; exact intraoperative IV fluid volume was unavailable, limiting definitive assessment.

Treatment of NPPE centers on relieving airway obstruction, restoring oxygenation, and providing positive-pressure respiratory support when necessary [1-4]. In this case, CPAP at 5 cmH₂O provided noninvasive positive-pressure support. A single intravenous dose of furosemide was also administered. The role of diuretic therapy in NPPE remains controversial because the primary pathophysiology is related to pressure-mediated pulmonary fluid translocation rather than necessarily systemic volume excess [2,3]. This distinction is clinically important because over-diuresis with orthostatic hypotension was reported in a recent appendectomy-associated NPPE case [6]. Accordingly, furosemide in the present case is best described as adjunctive therapy rather than obligatory treatment.

The renal abnormality was clinically important but mechanistically separate from the pulmonary complication. Imaging suggested pelviureteric junction stenosis with hydronephrosis, cortical thinning, and a large renal-pelvic calculus, while overall renal function remained preserved. Importantly, the CT report characterized the calculi as non-obstructing. These findings represent concurrent urologic pathology and should not be invoked as the cause of the acute pulmonary edema. The principal limitations of this report are the absence of the exact intraoperative intravenous fluid volume and a documented time to complete radiographic resolution.

## Conclusions

Post-extubation NPPE should be considered when acute respiratory deterioration follows laryngospasm or another form of upper-airway obstruction. In this patient, the immediate sequence of documented laryngospasm, severe hypoxemia, and new pulmonary opacities supported the diagnosis, with progressive improvement following positive-pressure respiratory support. Careful assessment for aspiration, cardiac dysfunction, and perioperative fluid overload remains important because these conditions may mimic NPPE. Concurrent abnormalities, such as unilateral pelviureteric junction pathology, should be evaluated independently rather than assumed to explain an acute perioperative pulmonary event.
